# Confocal analysis of nervous system architecture in direct-developing juveniles of *Neanthes arenaceodentata *(Annelida, Nereididae)

**DOI:** 10.1186/1742-9994-7-17

**Published:** 2010-06-16

**Authors:** Christopher J Winchell, Jonathan E Valencia, David K Jacobs

**Affiliations:** 1 Department of Ecology and Evolutionary Biology, University of California, Los Angeles, 621 Charles E. Young Drive South, Los Angeles, CA 90095-1606 USA; 2 Division of Biology, California Institute of Technology, 1200 East California Boulevard, MC 156-29, Pasadena, CA 91125 USA

## Abstract

**Background:**

Members of Family Nereididae have complex neural morphology exemplary of errant polychaetes and are leading research models in the investigation of annelid nervous systems. However, few studies focus on the development of their nervous system morphology. Such data are particularly relevant today, as nereidids are the subjects of a growing body of "evo-devo" work concerning bilaterian nervous systems, and detailed knowledge of their developing neuroanatomy facilitates the interpretation of gene expression analyses. In addition, new data are needed to resolve discrepancies between classic studies of nereidid neuroanatomy. We present a neuroanatomical overview based on acetylated α-tubulin labeling and confocal microscopy for post-embryonic stages of *Neanthes arenaceodentata*, a direct-developing nereidid.

**Results:**

At hatching (2-3 chaetigers), the nervous system has developed much of the complexity of the adult (large brain, circumesophageal connectives, nerve cords, segmental nerves), and the stomatogastric nervous system is partially formed. By the 5-chaetiger stage, the cephalic appendages and anal cirri are well innervated and have clear connections to the central nervous system. Within one week of hatching (9-chaetigers), cephalic sensory structures (e.g., nuchal organs, Langdon's organs) and brain substructures (e.g., corpora pedunculata, stomatogastric ganglia) are clearly differentiated. Additionally, the segmental-nerve architecture (including interconnections) matches descriptions of other, adult nereidids, and the pharynx has developed longitudinal nerves, nerve rings, and ganglia. All central roots of the stomatogastric nervous system are distinguishable in 12-chaetiger juveniles. Evidence was also found for two previously undescribed peripheral nerve interconnections and aspects of parapodial muscle innervation.

**Conclusions:**

*N. arenaceodentata *has apparently lost all essential trochophore characteristics typical of nereidids. Relative to the polychaete *Capitella*, brain separation from a distinct epidermis occurs later in *N. arenaceodentata*, indicating different mechanisms of prostomial development. Our observations of parapodial innervation and the absence of lateral nerves in *N. arenaceodentata *are similar to a 19th century study of *Alitta virens *(formerly *Nereis/Neanthes virens*) but contrast with a more recent study that describes a single parapodial nerve pattern and lateral nerve presence in *A. virens *and two other genera. The latter study apparently does not account for among-nereidid variation in these major neural features.

## Background

Mid 19th century studies of nereidid polychaetes were among the first to examine annelid nervous systems [1,2]. Since then, much has been learned about the neurobiology of adult annelids (reviews: [3-6]). With its capacity to effect various modes of movement (swimming, creeping, burrowing) and to integrate afferent signals from a diversity of cephalic and appendicular sense organs, the nereidid nervous system exhibits all the hallmarks of complex annelids, yet does not show exceptional specialization; its generalized morphology is traditionally considered archetypal within Annelida [3]. Furthermore, based on arguments that Aciculata, a modern polychaete clade including Nereididae, is most closely related to stem annelids [7,8], the nereidid nervous system may be little changed from an early, errant polychaete ancestor. Despite advances made in understanding adult annelid nervous systems, exceedingly few studies focus on their nervous system development, the notable exception being studies on leech (e.g., [9-11]).

As molecular systematists began to revolutionize views of animal phylogeny in the late 1990s (reviews: [12,13]; recent analyses: [14-16]), it became clear that the bilaterian superphylum Lophotrochozoa, which includes Annelida, has received less attention than the other bilaterian superphyla, Ecdysozoa and Deuterostomia. Knowledge of lophotrochozoan body plans and developmental processes is critical to reconstructing the morphologic, developmental, and genetic properties of the protostome-deuterostome ancestor [17-19]. Consequently, the Lophotrochozoa represent a major frontier for the field of evolutionary developmental biology (evo-devo).

The nereidid polychaete *Platynereis dumerilii *is currently a leading model in evo-devo studies of the bilaterian nervous system. With an emphasis on comparative developmental genetics, this species has been investigated for its photoreceptor organs and cell types [20,21], the early development of sensory-neurosecretory cell types in its brain [22], and the early neurogenesis of its trunk (primarily the ventral nerve cord) [23-25]. *P. dumerilii *and its confamilial *Alitta virens *(formerly *Nereis*/*Neanthes virens*) are the subjects of numerous other evo-devo studies, where gene expression patterns are often observed in the nervous system, but whose relations to specific neural structures are obscure. This is due in large part to insufficient knowledge of early life-stage internal anatomy. As noted by Ackerman et al. [26], careful description of the microanatomy of juvenile nereidids is greatly needed to corroborate and enhance studies of developmental gene expression. Furthermore, morphologic analysis with modern tools is needed to resolve long-standing discordances in the literature, such as the absence [27] or presence [28] of intersegmental lateral nerves among nereidids, and the conflicting descriptions of parapodial innervation presented in these studies.

The present study contributes to these objectives by analyzing the nervous system architecture in multiple juvenile stages of *Neanthes arenaceodentata*, a nereidid polychaete exhibiting direct development. The central approach used here, confocal laser scanning microscopy (CLSM) of immunolabeled acetylated α-tubulin, has proven useful in revealing (often in exquisite detail) the nervous system organization in widely diverse annelids (e.g., [29-34]). Because the microtubules of cilia and neuronal cell processes (axons and dendrites) are enriched with the acetylated isoform of α-tubulin [35,36], this method can be used to detect ciliated sense organs, and to produce comprehensive reconstructions of a nervous system's "wiring", although perikarya (neuronal cell bodies) are seldom labeled. To view the acetylated α-tubulin labeling in more informative contexts, the specimens analyzed here were also counterstained with a fluorescent nuclear label and in some cases with phalloidin, an F-actin label for muscle.

This study presents a comprehensive confocal overview of the nervous system in *N. arenaceodentata *juveniles. The results 1) reinforce much of the classic literature on adult nereidid neural architecture based on methylene blue staining, histologic reconstruction, electron microscopy, and neurophysiology; 2) reveal previously unknown peripheral nerve interconnections and aspects of parapodial muscle innervation; 3) promote the notion that Smith [28] overlooked variation in parapodial nerve architecture and the presence/absence of lateral nerves among his study taxa; and 4) determine the approximate stages of juvenile ontogeny by which key features of the cephalic nervous system become morphologically distinguishable. Examples of the latter include, first, portions of the stomatogastric system, consisting of the nerves and ganglia responsible for foregut innervation; second, the corpora pedunculata, prominent structures of the anterior brain thought to integrate information from the various cephalic sensory organs, and that are potential homologs of arthropod mushroom bodies [3,37]; and third, two pairs of ciliated sense organs: the chemosensory nuchal organs of the posterior head, and the poorly understood Langdon's organs of the anterior head.

## Results and Discussion

### Summary of juvenile development and external gross anatomy

Herpin [38] and Reish [39] described the basic aspects of reproduction and juvenile development of *Neanthes arenaceodentata*. Unlike many nereidids, reproductive individuals of this species do not undergo mass spawning as swarming epitokes, producing planktonic embryos that soon become ciliated larvae. Instead, mating individuals of this species form pairs that live together and spawn in a mucoid tube. The female dies shortly after spawning, and the male, capable of mating multiple times throughout life [40], broods his direct-developing clutch inside the tube for approximately 30 days (at ≈21°C). Growth and development are remarkably synchronous among clutch members. At about ten days post-fertilization, the embryos hatch from the egg capsules. Because no larval stage or metamorphosis occurs, these hatchlings are referred to as juveniles.

#### Hatchling, 3-, and 4-chaetiger stages

**Figure 1:**
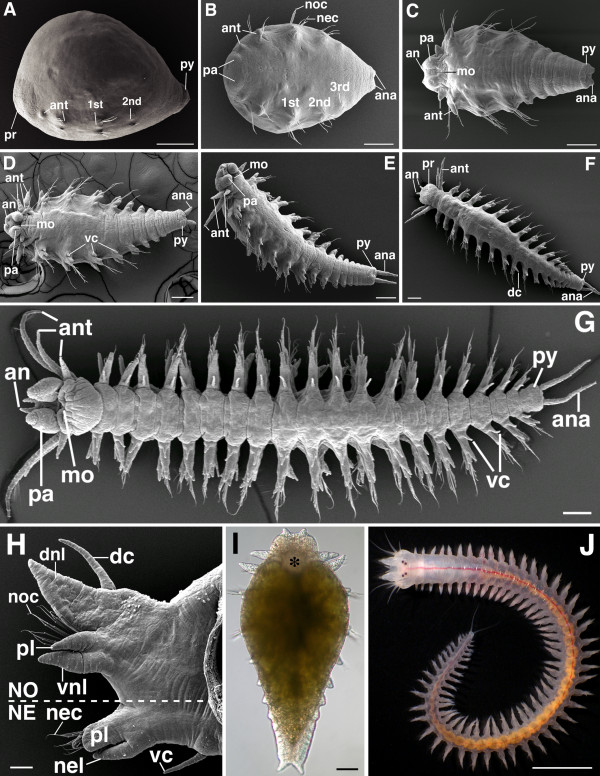
**SEM of juvenile developmental stages and basic external morphology of *N. arenaceodentata***. These worms were reared at ≈21°C, and all ages are approximate. Scale bars: **A-I **= 100 μm; **H **= 4 mm. Specimens processed for SEM experienced approximately 30% shrinkage. **A **is a lateral view, anterior to the left and ventral down; **B**, **C**, **D**, and **G **are ventral views, anterior to the left; **E **is a ventrolateral view, anterior to the upper left; **F**, **I**, and **J **are dorsal views with anterior up in **I, **and to the upper left in **F **and **J**. **A. **Hatchling, 10 days post-fertilization (dpf). **B. **Mid 3-chaetiger stage, 11 dpf. **C. **Late 3-chaetiger stage, 12 dpf. **D. **6 chaetigers, 15 dpf. **E. **9 chaetigers, 17 dpf. **F. **13 chaetigers, 22 dpf. **G. **20 chaetigers, 30 dpf. **H. **Transverse view (anterior side) of an adult parapodium taken from a mid-body segment. **I **and **J **are light micrographs. **I. **4 chaetigers, 13 dpf. The developing pharynx underlies the *asterisk*. **J. **Adult, 70 dpf. *1st, 2nd, 3rd *nascent parapodia, *an *antenna, *ana *anal cirrus, *ant *anterior cirrus, *dc *dorsal cirrus, *dnl *dorsal notopodial ligule, *mo *mouth, *NE *neuropodium, *nec *neurochaetae, *nel *neuropodial ligule, *NO *notopodium, *noc *notochaetae, *pa *palp, *pl *prechaetal lobe, *pr *prostomium, *py *pygidium, *vc *ventral cirrus, *vnl *ventral notopodial ligule.

The presence of two or three chaetigers (chaetae-bearing segments), a slightly elongated posterior end bearing the pygidium, and a distinct anterior mound (the nascent prostomium) characterize the hatchling stage (Fig. 1A). Several small, ventrolateral protuberances are also present. These correspond to the sensory first and second anterior cirri (the second of which is chaetigerous), and to the first and second parapodia. The prostomium and anterior cirri ultimately form the head (see below). Four large yolk-filled macromeres fill the hatchling and give it a humpback appearance. These cells stop dividing after the third embryonic cleavage, become enclosed within the digestive tract, and are expended as a food source during juvenile development [39]. By the mid 3-chaetiger stage (Fig. 1B), sensory feeding palps, located ventrally on the prostomium, begin to take shape, and the sensory anal cirri appear as small knobs on the pygidium. By the late 3- and 4-chaetiger stages (Fig. 1C, I), the prostomium has enlarged significantly and bears emerging antennae at its anterior terminus. Behind the prostomium, a definite mouth has formed on the ventral surface. The anus appears as a cleft between the anal cirri, and the initial, achaetigerous developmental phase of several parapodial pairs can be distinguished in the posterior growth zone anterior to the pygidium.

#### Further juvenile development

Parapodial lengthening, a relatively continuous rate of posterior segment addition, and changes in the anterior cirri characterize the next stages of juvenile development (Fig. 1D-F). In 6-chaetiger juveniles (Fig. 1D), the third (anteroventral) anterior cirri begin to emerge below the first (anterodorsal) ones, and by the 13-chaetiger stage (Fig. 1F), the second (posterodorsal) anterior cirri cephalize, changing from chaetigerous parapodia-like structures (Fig. 1C) to long head appendages lacking chaetae. Development of the fourth (posteroventral) anterior cirri was not observed in any juvenile stage examined here, but they are present in adults below the second cirri. Cephalization of larval/juvenile anterior parapodia is typical of nereidids and related families (e.g., Hesionidae, Chrysopetalidae) [41]. In nereidids, this process forms a post-prostomial ring of tissue, often referred to as the peristomium, bearing all anterior cirri. The peristomium is traditionally defined as an anterior, pre-segmental body region developmentally distinct from the trunk segments behind it. However, Ackermann et al. [26] show that the anterior cirri of *P. dumerilii *arise from the same embryonic blastomeres that produce the segmented trunk. Given this finding, it seems safe to conclude that, first, two highly modified and fused segments bear the anterior cirri; second, the ganglion shared by each pair of anterior cirri (in adults, there is an anterior and posterior pair on each side) is serially homologous to the parapodial ganglia of the more typical trunk segments [42]; and third, the bi-segmented ring carrying the anterior cirri in nereidids should no longer be called the peristomium, "achaetous ring" [43] should instead be used. Whether nereidids retain any trace of a peristomium remains to be answered (see Ackermann et al. [26] for more on this controversy); some authors consider it limited to lips, the ventral epidermis surrounding the mouth [41].

#### 20-chaetiger and older stages

At approximately 20 chaetigers (Fig. 1G), juveniles disperse from the parental tube and begin feeding. By this stage only one or two pre-pygidial segments exhibit parapodial morphogenesis, whereas earlier stages exhibited a growth zone with at least five pre-pygidial morphogenetic segments (Fig. 1E). Fig. 1J shows an adult specimen, and Fig. 1H shows an isolated parapodium. Parapodia have two main divisions, a dorsal notopodium and a ventral neuropodium. Each of these is further divided into smaller processes: the dorsal and ventral cirri are the main parapodial sensory structures; the ligules are highly vascularized gill-like structures, also apparently capable of sensation; and chaetae (bristles) project from between the pre- and post-chaetal lobes. Embedded between these lobes is a chaetal sac, from which the chaetae develop and are basally anchored [44]. During chaetal movement (specifically muscle-controlled protraction and retraction), the chaetal sac glides along the aciculum [45], a stiff internal support rod originating at the proximal base of each parapodial division and tapering to a fine point in the pre-chaetal lobe.

### CLSM overview of hatchlings

**Figure 2:**
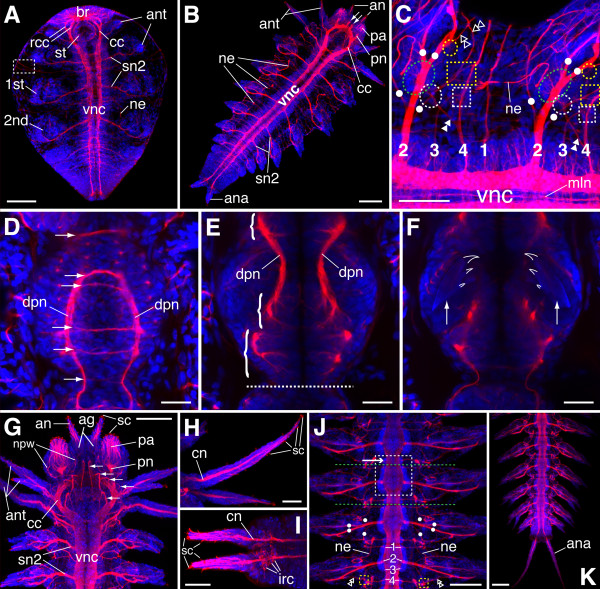
**Various components of the juvenile nervous system of *N. arenaceodentata***. Acetylated α-tubulin immunoreactivity is red; cell nuclei are blue. Scale bars = 100 μm in **A**, **B**, **G**, **J**, **K**; 50 μm in **C**, **H**, **I**; 25 μm in **D**-**F**. **A. **Hatchling; ventral view, anterior to the top. Segmental nerves 1 and 4 are visible within the *dashed box*. **B. **5-chaetiger juvenile; ventral view, anterior to the upper right. *Arrows *point to the first (medial-most) pair of stomatogastric nerves. **C. **9-chaetiger juvenile; ventrolateral view between chaetigers 5 and 6, anterior to the left. *Numerals 1-4 *refer to segmental nerves 1-4. *Dashed circles *and *dashed boxes *enclose regions expected to contain nerve interconnections described in the text. *Dashed green ellipses *represent parapodial ganglia; *closed double arrowheads *point to axons of the sn2/4 interconnection; *dots *are positioned near the bases of the four main parapodial nerve branches, pn1-4 (in order from anterior to posterior); and *open double arrowheads *point to nerves with uncertain dorsal termini. **D**-**F. **Contiguous Z-projections of the dorsal portion of a 9-chaetiger juvenile's pharynx, anterior to the top. **D. **Dorsal-most Z-projection (above the pharyngeal lumen). *Arrows *point to commissural nerves, and punctate labeling probably represents innervation of pharyngeal muscle. **E. **Middle Z-projection. *Brackets *contain regions of the main dorsal pharyngeal nerves that show fine neurite connections to cells of presumed ganglia. The *dashed line *separates the pharynx from the esophagus. **F. **Ventral-most Z-projection. *Arrows *point to the bases of the developing jaws, the tips of which are outlined for clarity. **G. **12-chaetiger juvenile anterior end; ventral view, anterior to the top. *Arrows *point to stomatogastric nerves 1-5 (anterior to posterior). **H. **Anterior cirri of a 12-chaetiger juvenile (left side of head). The anterodorsal cirrus is at the bottom of the panel; the posterodorsal cirrus is above the latter. **I. **Anal cirri of an 8-chaetiger juvenile; ventral view. **J, K. **20-chaetiger juvenile; ventral view, anterior to the top. **J. **Mid-body segments. A single pair of VNC ganglia is *boxed*, and an *arrow *points to its pre-septal portion. *Dashed green lines *delimit a single segment. All other labeling (zoom in to see) follows panel **C**. **K. **Posterior end. *1st/2nd *nascent parapodia, *ag *antennal ganglion, *an *antenna, *ana *anal cirrus, *ant *anterior cirrus, *br *brain, *cc *circumesophageal connective, *cn *cirrus nerve, *dpn *dorsal pharyngeal nerve, *irc *immunoreactive cell, *mln *median longitudinal nerve of the vnc, *ne *nephridium, *npw *nerve of palp wall, *pa *palp, *pn *axial palp nerve, *rcc *root of the circumesophageal connective, *sc *sensory cilia, *sn2 *segmental nerve 2, *st *stomodeum, *vnc *ventral nerve cord.

Because *N. arenaceodentata *embryos lack ciliation [39], and because no residual trochophore-specific neuronal elements (apical ganglion, larval eyes, circumferential nerve rings) were detected in hatchlings, it appears that this species, having evolved a derived mode of development, has concomitantly lost all essential larval features of its ancestors. Consistent with this, the major neural features observed in hatchlings reflect the basic organization of the adult nereidid nervous system. In particular, the central nervous system (CNS) is well developed, with a large anterior brain joined to the two prominent tracts of the ventral nerve cord (VNC) via circumesophageal connectives (CCs) (Fig. 2A). Key parts of the peripheral nervous system (PNS), most notably the 2nd segmental nerves, are also well developed. These nerves link the anterior cirri and parapodia (both of which appear as dense, ventrolateral cell clusters) to the VNC. Other, less conspicuous peripheral nerves observed at this stage include the 1st and 4th segmental nerves, which are rooted in the VNC and course laterally between the segmental appendages (Fig. 2A). In addition, a nerve ring with bilateral roots in the brain circles the stomodeum (Fig. 2A) and appears to be the first component of the stomatogastric nervous system to develop.

Other confocal analyses of developing polychaetes show that major components of the adult nervous system begin to form during larval life, before metamorphosis occurs. For example, late larvae of the serpulid *Pomatoceros lamarckii *[46] and the sabellariid *Sabellaria alveolata *[47] have rudimentary brains, CCs, VNCs, and segmental peripheral nerves. Therefore, despite *N. arenaceodentata's *derived developmental mode, its hatchling nervous system exhibits major features typical of larval polychaetes.

In addition to the nervous system, the cilia of developing nephridia are immunoreactive to acetlylated α-tubulin and are visible in the third chaetiger (the segment bearing the second pair of parapodia) of hatchlings (Fig. 2A). These excretory organs are more apparent in later stages (Fig. 2B, C, J). The anterior funnels of the nephridia are embedded in the intersegmental septum between the 4th and 1st segmental nerves of adjacent segments, and their ducts meander posteriorly to discharge filtrate anterior to the parapodial bases [48].

### Sensory appendages

**Figure 3:**
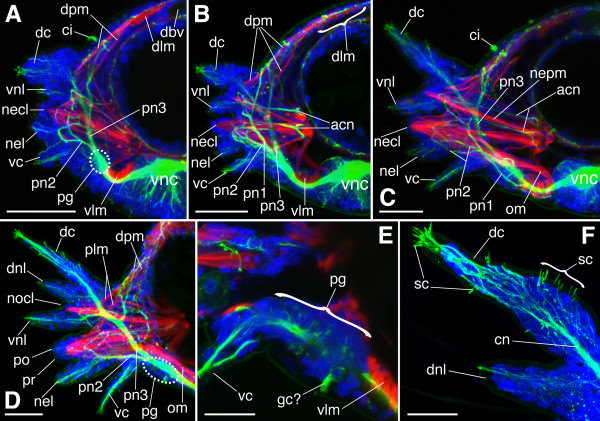
**Parapodial innervation and developmental sequence of parapodial processes in *N. arenaceodentata***. The parapodia shown were isolated from different 13-chaetiger juveniles. Panels **A**-**D **are Z-projections of nearly entire parapodia; fluorescent signal from deep structures is muted. Acetylated α-tubulin immunoreactivity is green; labeling of F-actin is red; cell nuclei are blue. Scale bars = 50 μm in **A**-**D**; 25 μm in **E**, **F**. **A. **15th parapodium (pre-chaetigerous), anterior aspect. This Z-projection excludes anterior optical sections that show pn1. **B. **13th (youngest chaetigerous) parapodium, anterior aspect. **C. **12th parapodium, anterior aspect. **D**-**F. **3rd parapodium, posterior aspect. **D. **Entire parapodium. **E. **Close-up of parapodial ganglion; Z-projection of 5 internal optical sections. The putative neuronal growth cone (*gc?*) is tipped with red F-actin labeling (zoom in to see). **F. **Close-up of dorsal cirrus. *acn *acicula-associated nerve, *ci *ciliary tuft, *cn *cirrus nerve, *dbv *dorsal blood vessel, *dc *dorsal cirrus, *dlm *dorsal longitudinal muscle, *dnl *dorsal notopodial ligule, *dpm *dorsal parapodial muscle, *necl *neuropodial chaetal lobe, *nel *neuropodial ligule, *nepm *neuropodial protractor muscle, *nocl *notopodial chaetal lobe, *om *oblique muscle, *pg *parapodial ganglion, *plm *parapodial levator muscle, *pn1*/pn2/*pn3 *1st/2nd/3rd branch of the parapodial nerve, *po/pr *post- and pre-chaetal lobes of the neuropodium, *sc *sensory cilia, *vc *ventral cirrus, *vlm *ventral longitudinal muscle, *vnc *ventral nerve cord, *vnl *ventral notopodial ligule.

**Figure 4:**
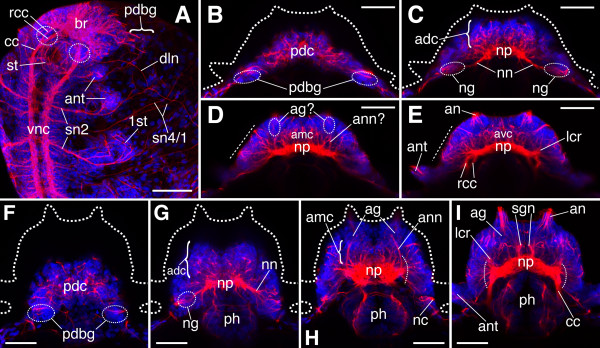
**Overview of the cephalic nervous system in hatchlings, late 3-, and 5-chaetiger juveniles of *N. arenaceodentata***. Acetylated α-tubulin immunolabeling is red; cell nuclei are blue. Scale bars = 100 μm in **A**; 50 μm in **B**-**I**. **A. **Hatchling; ventrolateral view, anterior to the top. *Dashed ovals *approximate the locations of the developing palps. **B**-**I **are dorsal views of heads, anterior to the top. **B**-**E. **Contiguous Z-projections (dorsal-most to ventral-most) of a late 3-chaetiger juvenile. In **B**, positions of the PDBGs are inferred from panel **A**. In **D **and **E**, *dashed lines *indicate densely innervated regions of the lateral prostomium, where developing brain ganglia and sensory cells reside. **F**-**I. **Contiguous Z-projections (dorsal-most to ventral-most) of a 5-chaetiger juvenile. *Dashed arcs *in **H **and **I **indicate the numerous roots of cephalic nerves in the lateral neuropile; many are presumed to be tegumentary nerve roots. *1st *nascent parapodium,* adc *anterodorsal cortex of brain, *ag *antennal ganglion, *amc *anteromedian cortex of brain, *an *antenna, *ann *antennal nerve, *ant *anterior cirrus, *avc *anteroventral cortex of brain, *br *brain, *cc *circumesophageal connective, *dln *dorsolateral longitudinal nerve, *lcr *lateral common root of numerous cephalic nerves, *nc *nuchal organ cilia, *ng *nuchal ganglion, *nn *nuchal nerve, *np *neuropile, *pdc *posterodorsal cortex of brain, *pdbg *posterodosal brain ganglion, *ph *pharynx, *rcc *root of a circumesophageal connective, *sgn *stomatogastric nerve (of the first pair), *st *stomodeum, *sn1/2/4 *segmental nerves 1/2/4, *vnc *ventral nerve cord.

**Figure 5:**
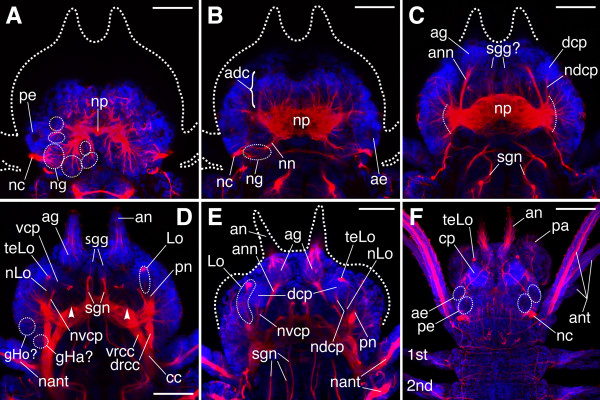
**Overview of the cephalic nervous system in older *N. arenaceodentata *juveniles**. **A**-**D. **Contiguous Z-projections (in order from dorsal-most to ventral-most) of a 9-chaetiger juvenile head. The *dashed ellipses *in **A **correspond to cell clusters at the terminal ends of presumed axonal tracts, and thus probably represent posterior brain ganglia. *Dashed curves *in **C **indicate roots in the lateral neuropile of presumed tegumentary nerves. *Arrowheads *in **D **indicate the roots of the second stomatogastric nerves. **E. **13-chaetiger juvenile head. The optical sections comprising this Z-projection (located ventrally in the head) were selected to highlight the distinct paths of cephalic nerves leading to the dorsal and ventral masses of the corpora pedunculata. **F. **20-chaetiger juvenile anterior end; nearly full Z-projection showing superficial features. The corpora pedunculata (cp) are evident by their intense nuclear staining. *1st, 2nd *parapodia,* adc *anterodorsal cortex of the brain, *ae *anterior eye, *ag *antennal ganglion, *an *antenna, *ann *antennal nerve, *ant *anterior cirrus, *cc *circumesophageal connective, *cp *corpora pedunculata, *dcp *dorsal mass of the corpora pedunculata, *drcc *dorsal root of cc, *gHa? *presumed Hamaker's commissural ganglion, *gHo? *presumed Holmgren's cerebral commissural ganglion, *lcr *lateral common root of several cephalic nerves, *Lo *Langdon's organ, *nant *nerve of anterior cirrus, *nc *nuchal organ cilia, *ndcp *nerve of the *dcp*, *ng *nuchal ganglion, *nLo *nerve of Langdon's organ, *nn *nuchal nerve, *np *neuropile, *nvcp *nerve of the *vcp*, *pa *palp, *pe *posterior eye, *pn *base of axial palp nerve, *sgg *stomatogastric ganglion, *sgn *stomatogastric nerve, *teLo *terminal endings of sensory-cell peripheral processes from Langdon's organ, *vcp *ventral mass of the corpora pedunculata, *vrcc *ventral root of cc.

By the 5-chaetiger stage, the cephalic appendages (antennae, palps, anterior cirri) are richly innervated, and the axial palp nerves, composed of sensory fibers from the palp tips, have clearly formed in the ventral brain (Fig. 2B). The cephalic appendages, as well as the anal and parapodial cirri, are generally regarded as chemoreceptors, and they are all related in adult nereidids by their expression of a similar sensory morphology. They bear many multiciliate penetrative bipolar sensory neurons, the most common receptor type among annelids [49]. Along with their associated glia, multiple receptors are grouped into small sensory organs on each appendage [50-52]. Each organ's bundled peripheral processes (dendrites) terminate in short tufts of sensory cilia that penetrate the overlying cuticle. The central processes (axons) of an organ are also bundled, and as they travel proximally toward the CNS, they usually converge with the central processes of other sensory organs to form a discrete nerve at the appendage base. Our observations did not resolve all grouped cells of the appendicular sense organs, but sensory cell processes, including the epicuticular ciliary tufts, are easily detected in juvenile palps and antennae (Fig. 2B, G), anterior cirri (Fig. 2H), anal cirri (Fig. 2I), and parapodial cirri (Fig. 3D, F). In the case of the antennal sense organs, bundled central processes pass into a ganglion at the antennal base, and the antennal nerve forms caudal to this ganglion (Fig. 4I and 5C, E). Note that numerous acetylated α-tubulin immunoreactive cells in the pygidium cluster around the base of each anal cirrus nerve (Fig. 2I); the significance of these cells is uncertain.

### Segmental nerve roots and position of VNC ganglia

A close inspection of the roots of the segmental nerves (where they connect to the VNC) in 9-chaetiger juveniles (Fig. 2C) shows a pattern identical to that described for the adults of three nereidid genera (*P. dumerilii*, *A. virens*, and *Hediste diversicolor*) by Smith [28]. Each trunk segment bears four main pairs of peripheral nerves that are numbered 1-4 (from anterior to posterior), and each pair provides sensory and motor innervation to non-overlapping portions of the segment [28]. The 2nd segmental (parapodial) nerve, by far the largest of the four, alone provides direct parapodial innervation. The position of VNC ganglia in nereidids is out of register with the trunk segments [27,28]; consequently, the anterior-to-posterior order of segmental nerves depends on whether a segmental or a ganglionic perspective is taken. To clarify, the anterior portion of each ganglion is pre-septal, and because this portion bears the 4th segmental nerve, the nerve order for each ganglion is 4, 1, 2, 3, whereas for each segment it is 1, 2, 3, 4. This distinction is most easily made in 20-chaetiger juveniles; by this stage the VNC has become clearly ganglionated (Fig. 2J, K). In addition, the VNC contains a median longitudinal nerve (Fig. 2C), considered part of the annelid groundplan [53]. This nerve was observed under high magnification in *N. areanaceodentata *juveniles through the 20-chaetiger stage (data not shown), but may be lost during later ontogeny, as has been reported for *A. virens *[54].

### Absence of lateral nerves

Smith's [28] reconstruction of the nereidid nervous system includes a pair of "lateral nerves". These two nerves run along opposite outer edges of the ventral longitudinal muscle bands and connect the 3rd segmental nerves of successive segments (note that they are not equivalent to the peripheral longitudinal nerves discussed by Orrhage and Müller [53]). He classified the 3rd segmental and lateral nerves as primarily proprioceptive (i.e., they presumably sense stretch in the ventral longitudinal muscles), and he hypothesized the importance of their intersegmental connections in facilitating the suprasegmental rhythm of body undulation required for efficient swimming. Similarly, Quatrefages [2], studying the nereidid *Eunereis longissima*, described a segmental nerve that is rooted in the VNC ganglion of one segment and penetrates the intersegmental septum to make a neural connection in the preceding segment.

Whether the lateral/intersegmental nerves described by Smith [28] and Quatrefages [2] are homologous is uncertain, but their presence suggests at least a common neuroarchitectural theme in some nereidids. However, Hamaker [27] was adamant about the absence of such nerves based on his examination of *A. virens*. Moreover, the present analysis of *N. arenaceodentata *juveniles finds no compelling evidence for any trans-septal peripheral nerve linking all 3rd segmental nerves. The apparent presence of lateral nerves in some nereidids and absence in others suggests among-nereidid variation in a major neural feature. But what could explain the opposing findings of Hamaker [27] and Smith [28] regarding the absence/presence of lateral nerves in *A. virens*? It is possible that Smith's [28] reconstruction of nereidid lateral nerves — and of parapodial innervation (see below) — is representative of only *P. dumerilii*, which claimed the lion's share of his attention (his study, bearing on many aspects of segmental innervation, incorporated 150 preparations of *P. dumerilii *but only 30 of *H. diversicolor *and a mere 10 of *A. virens*). Further investigation is necessary to substantiate the possible variation in this character, assess its phylogenetic utility, and determine whether other neural adaptations are in place (such as a CNS pathway connecting 3rd segmental nerves) to account for the absence of lateral nerves.

### Peripheral nerve interconnections

Within each segment, Smith [28] also identified two types of interneurons of the PNS, each type connecting a different pair of segmental nerves: the 2nd and 4th, and the 3rd and 4th. Confocal analyses of *N. arenaceodentata *juveniles revealed these same interconnections. First, the interneuron (interneurons?) connecting the 2nd and 4th segmental nerves resides in a cell cluster immediately posterormedial to the parapodial ganglion (Fig. 2C, dashed white circles). A short dendrite (dendrites?) emerges from this cell cluster and extends anteriorly toward the 2nd nerve, while a long axon (axons?) extends posteromedially to meet the 4th nerve. In forming the second interconnection (between the 3rd and 4th segmental nerves), the 3rd nerve extends laterally from the VNC and gradually arcs posteriorly toward the 4th nerve. Where the two meet, the 3rd nerve terminates in a cell cluster, presumably synapsing with the interneurons making the connection (Fig. 2C, dashed white boxes). Smith [28] hypothesized that for both interconnections, excitation is transmitted to the 4th segmental nerve, which is likely the primary conduit of motor impulses to the dorsal and ventral longitudinal muscles. Corroborating the latter notion, Wilson [55] recorded action potentials in *Neanthes brandti*'s dorsal longitudinal muscle upon electrical stimulation of the 4th segmental nerve. Thus, activity of the longitudinal muscles (which contract to generate body flexure) may be adjusted by efferent signals related to parapodial orientation (from the 2nd/4th-nerve interconnection) and the degree to which the ventral longitudinal muscle is stretched (from the 3rd/4th-nerve interconnection). This coordination of parapodial movement and body flexure is likely necessary to achieve finely controlled ambulation and swimming [28].

Two other peripheral nerve interconnections were observed that, to the authors' knowledge, have not previously been described. The first is a small plexus (Fig. 2C and 2J, dashed yellow boxes) located ventrolaterally in each segment, just dorsolateral to the 3rd/4th segmental nerve interconnection. This plexus may serve to integrate fibers from three different nerves: the 4th segmental nerve, pn4 (the 4th, posterior-most branch of the parapodial nerve), and a nerve that extends from an uncertain dorsal location. This latter nerve appears to contact the posterior set of dorsal parapodial muscles or has a connection with pn3 in that vicinity (data not shown). The cell-body locations and specific identities of the neurons interacting here are as yet unknown; there may also be an association with fibers of the 2nd/4th segmental nerve interconnection (Fig. 2C, within dashed white circles), potentially increasing the complexity of this plexus. The second previously undescribed interconnection (Fig. 2C, dashed yellow circles) occurs between the proximal end, posterior side of pn3 and another nerve with an undetermined dorsal terminus (although it appeared to course dorsomedially a short distance beyond the dorsoventral midpoint of the body, where it connected to muscle or another nerve [data not shown]). We infer this as a connection between two separate nerves, as opposed to a branch point of pn3, because a small node of cells appears to couple the intersecting fibers. Further research is needed to confirm this supposition. The two nerves with undetermined dorsal termini contributing to the aforementioned peripheral interconnections cross paths just posterior to the parapodium, at an approximately median location along the dorsoventral body axis (Fig. 2C).

### Stomatogastric nerves and pharyngeal innervation

The stomatogastric nerves, which equip the alimentary canal (primarily the pharynx) with motor and sensory innervation, are fundamental components of annelidan nervous systems [3,53]. The presence of five pairs of stomatogastric nerves rooted in the ventral brain (1st and 2nd pairs) and CCs (3rd, 4th, and 5th pairs) is characteristic of nereidids [56]. In *N. arenaceodentata *juveniles, the 1st (medial-most) pair of stomatogastric nerves develops by the 5-chaetiger stage (Fig. 2B), and the remaining four pairs develop by the 12-chaetiger stage (Fig. 2G). Nerves within the pharynx itself are well developed by the 9-chaetiger stage (Fig. 2D-F). A pair of longitudinal pharyngeal nerves runs along the dorsal side of the pharynx, continuing into the esophagus. Along the way, several commissures connect the left and right longitudinal nerves (Fig. 2D). The presence of these commissures, coupled with the arrangement of fine nerve fibers projecting into the pharyngeal tissue (Fig. 2E), indicates the existence of one anterior and possibly two posterior pharyngeal ganglia. The close proximity of the two putative posterior ganglia, which occur posterior and medial to the jaws (Fig. 2F), suggests they could instead be one large ganglion. In support of this, Henry [57], who examined the basic pharyngeal innervation of several polychaetes, diagrammed one very large ganglion behind each jaw in *A. virens*; she also determined that only the 2nd and 3rd stomatogastric nerves (Fig. 2G, second and third arrows down) terminate at these ganglia. Apart from this information (to the authors' knowledge), no detailed analysis of the stomatogastric nervous system of a nereidid has been published. Brief examination of the ventral pharyngeal innervation in *N. arenaceodentata *juveniles suggests a general pattern similar to the dorsal pharynx. In addition, commissures of the dorsal longitudinal nerves connect to commissures of the ventral longitudinal nerves, forming more-or-less continuous nerve rings. Pharyngeal nerve rings are known from a diversity of other polychaetes (nephtyids, eunicids, aphroditids, phyllodocids, and capitellids) [3,58], suggesting such structures were present in the annelid stem species.

### Developmental sequence of parapodial processes

The 15th parapodium of a 13-chaetiger juvenile is the earliest stage of parapodial development reported here (Fig. 3A). The 16th and 17th parapodia are also present (closer to the pygidium) in these juveniles, but were difficult to dissect from the body, which is less than two millimeters long. The 15th parapodium already exhibits five processes. From dorsal-to-ventral, these are: dorsal cirrus, ventral notopodial ligule, neuropodial chaetal lobe, neuropodial ligule, and ventral cirrus (Fig. 3A). Of these, the middle three are smaller and less distinct than the dorsal and ventral cirri, suggesting the cirri develop first. This is incongruent with other nereidids [59-61] but not surprising, as no two nereidids are yet known to share the same developmental sequence of parapodial processes [61]. This incongruence may reflect a methodological difference, as the analyses done here involve progressively older parapodia (from posterior to anterior) in a single ontogenetic stage (13-chaetigers), whereas previous studies analyze the same parapodium (one of the most anterior ones) in progressively older larvae/juveniles.

The least developed parapodium displaying emerged chaetae is the 13th. Neither it nor the 12th exhibit newly developed processes, but internal differentiation of the notopodial chaetal lobe is indicated by the development of muscles and nerves (Fig. 3B, C; see below). The 3rd parapodium shows significant changes (Fig. 3D). Namely, the dorsal notopodial ligule has developed and grown to nearly the same size as most other processes, and pre- and post-chaetal portions of the neuropodial chaetal lobe are present. The notopodial chaetal lobe, in contrast, is just beginning to emerge. The late formation of the dorsal notopodial ligule, which ultimately becomes the largest parapodial process (Fig. 1H), followed by the even later emergence of the notopodial chaetal lobe, is consistent with parapodial development in *A. virens *[60], suggesting a phylogenetic signal in the developmental sequence of parapodial processes.

### CLSM analysis of parapodial innervation

#### Parapodial nerve branch 1 (pn1)

Nervous impulses are conducted between the VNC and each parapodium via the parapodial (2nd segmental) nerve, which, at the base of the parapodium, passes into a parapodial ganglion. These ganglia, known from most errant polychaete families [3], are prominent components of the PNS, and reside, at least in nereidids, between the ventral longitudinal muscle and the base of the ventral cirrus (Fig. 2C and 3A, D, E). In *N. arenaceodentata *juveniles, each parapodial nerve divides into four main branches (dots in Fig. 2C, J). These branch points are characterized as either preganglionic or postganglionic with respect to the parapodial ganglion. The parapodial nerve's first (most anterior) branch, pn1, is preganglionic (arising proximal to the ganglion) and ascends the anterior face of the parapodium (Fig. 3B, C). About halfway up, it divides into three smaller branches: the largest continues dorsomedially and terminates at the dorsal parapodial muscles, whereas the other branches, which are minute, appear to terminate distally in the developing notopodial chaetal lobe, possibly in chaetal sac musculature or at a peripheral connection shared with pn3 (see below).

#### Parapodial nerve branch 2 (pn2)

The course of the parapodial nerve's second branch, pn2, which innervates only the neuropodium, is similar to Hamaker's [27] description of the same nerve found in *A. virens*. A short distance beyond the parapodial ganglion, pn2 divides into anterior and posterior branches, both of which proceed distally along the ventral side of the neuropodial chaetal lobe (Fig. 3A, B, C). The neuropodial ligule receives fibers from the posterior branch (Fig. 3D). Approximately midway along the neuropodial chaetal sac, both the anterior and posterior branches of pn2 turn abruptly back toward the midline. Hamaker's [27] description of pn2 goes no further, but he probably could not trace it beyond this point, as its dark staining would be difficult to detect against the opaque neuroaciculum. However, the analyses done here reveal one very conspicuous branch of pn2 that courses backward to the very base of the neuropodial chaetal lobe, roughly paralleling the neuroaciculum. This acicula-associated nerve terminates on muscles originating at the acicular head (Fig. 3B, C), one of which inserts at the tip of the notopodium (Fig. 3C) and has been described in *H. diversicolor *as a neuropodial protractor [45]. During creeping, this muscle permits full neuropodial extension, an action that enhances the parapodium's backward power stroke to push the segment forward. Although not observed here, other important features of pn2 can be inferred from parapodial analyses of other polychaetes. In the polynoid *Harmothoe *[62] and the nereidids *A. virens *and *H. diversicolor *[51], neuropodial bristle receptors with finely branching dendrites lie directly on the chaetae. Their ability to sense chaetal movement has been demonstrated by the recording of action potentials in the parapodial nerve upon gentle touch to any neurochaeta [51,62]. Additionally, multiple bipolar stretch receptors reside in the ventral neuropodial wall of *Harmothoe *[62], and in the anterior and posterior neuropodial walls of *A. virens *and *H. diversicolor *[51]. Dorsett's [51] drawing of the nereidid neuropodial bristle and stretch recpetors shows their axons joining separate ventral parapodial nerves; these are interpreted here as the anterior and posterior branches of pn2. Further work to detect bristle and stretch receptors using antibodies against certain neurotransmitters is merited. For example, the use of serotonin antibodies revealed chaetal sac neurons in sabellariid larvae [47], although it is unknown whether these are sensory or motor.

#### Parapodial nerve branch 3 (pn3)

The parapodial nerve's largest branch, pn3, arises from the parapodial ganglion, ascends the posterior wall of the parapodium, and in general divides and follows paths similar to those described for the same nerve in *A. virens *[27]; CLSM analysis is nevertheless useful in elaborating its anatomy. At the level of the notopodium, a branch from pn3 extends dorsomedially and, like the dorsomedial branch of pn1, terminates at the dorsal parapodial muscles (Fig. 3A-D) (the difference is that pn1 and pn3 go to anterior and posterior sets of these muscles, respectively). Like the neuropodial protractor and other acicular muscles, the dorsal parapodial muscles function in creeping. They are most important during the preparatory stroke, pulling the parapodium inward, upward, and forward before the backward-deflecting power stroke [45]. pn3's dorsomedial branch may also terminate on the parapodial levator (Fig. 3D), a muscle that originates at the dorsal parapodial wall and inserts via separate bundles on the notopodial and neuropodial chaetal lobes. This muscle lifts the parapodium from the substrate while pulling in its tips, and is thus another important player during the preparatory stroke [45]. Above the levator and dorsal parapodial muscles, tufts of cilia were sometimes observed emanating from the outer body wall (Fig. 3A, C). The nature of the cells bearing these cilia is uncertain. No neural connections to them were evident, suggesting they are not sensory; they may instead serve to move gas-exchanging water currents over capillaries that reside in this region of the integument [63].

Laterally, pn3 branches separately to each ligule of the notopodium (Fig. 3D). In anterior aspects of the parapodia, and just medial to the ventral ligule, a long straight nerve running parallel to the acicula-associated nerve of the neuropodium courses medially to muscles surrounding the notopodial acicular head (Fig. 3B, C). The origin of this notopodial acicula-associated nerve is uncertain. It may be a branch from pn3 that projects anteriorly toward the lateral branches of pn1 before turning medially toward the acicular head. However, individual confocal optical sections (rather than the Z-projections presented in Fig. 3B and 3C) hint at the possibility of a multicellular node, near the base of the ventral notopodial ligule, linking certain lateral branches of pn1 and pn3, the notopodial acicula-associated nerve, and ligular nerve fibers (data not shown). This node may represent the notopodial ganglion observed by Henry [57], but further investigation is needed to confirm its presence and to resolve the spatial relations among these nerves. pn3 receives the sensory nerve of the dorsal cirrus (Fig. 3D, F); in contrast, sensory fibers from the ventral cirrus do not fasciculate into a single nerve. Instead, many fine fibers and several larger bundles independently extend into the parapodial ganglion (the Z-projection of Fig. 3E shows two of the larger bundles). Some nerve fibers of the ventral cirrus may also combine with pn2 outside of the parapodial ganglion, as observed in *A. virens *[27]. Other components of pn3 not observed here but whose presence is inferred from analyses of other nereidids [51] include three mechanoreceptor types: notopodial bristle receptors, a notopodial flap receptor (a large tripolar neuron residing in the dorsal notopodial ligule, sensing its flexure during locomotion), and a dorsal cirrus receptor (a bi- or tripolar neuron residing below the base of the dorsal cirrus, sensing its side-to-side, muscle-controlled movement).

#### Parapodial nerve branch 4 (pn4)

As described above, pn4 is the posterior-most branch of the parapodial nerve; it also has the proximal-most branch point along the parapodial nerve, diverging slightly before pn1, the only other preganglionic branch (the other branches, pn2 and pn3, diverge distal to the parapodial ganglion) (Fig. 2C, J and Fig. 3A-D). Unlike the other parapodial-nerve branches, pn4 barely enters the parapodium; it instead passes posterolaterally into the peripheral plexus described above.

#### Among-nereidid variation in parapodial innervation

In Smith's [28] reconstruction of the nereidid pattern of parapodial innervation, he apparently overlooked variation that is present among his three study taxa: *P. dumerilii*, *H. diverisicolor*, and *A. virens*. He presented a single pattern in which there are five main branches of the parapodial nerve: two that ascend the anterior parapodial face (first and second branches), one restricted to the underside (third branch), one represented by the central fibers of the ventral cirrus sensory organs (fourth branch), and one that ascends the posterior face (fifth branch). This contrasts with the pattern observed in *A. virens *by Hamaker [27], which is very similar to the pattern observed here for *N. arenaceodentata*. The basic differences are that in *N. arenaceodentata *and *A. virens*, only one nerve, pn1, ascends the anterior parapodial face, and no pn4-equivalent is present in Smith's [28] reconstruction. Furthermore, Dorsett's [51] diagram showing basic aspects of parapodial innervation in *A. virens *and *H. diversicolor *is consistent with the pattern observed here in that his second branch, like pn2, innervates the neuropodium, not the anterior parapodial face. Despite these differences, Smith's [28] third and fourth branches have a distribution similar to pn2, and his fifth branch appears to be equivalent to pn3.

### CLSM analysis of the cephalic nervous system

#### Hatchling and late 3-chaetiger stages

With the general labeling strategy employed here, the hatchling brain appears as a compact mass of neuronal cell bodies and processes. Its chief attributes are a dense region of ventral commissures occurring between the roots of the CCs (Fig. 2A), and three bush-like groupings of processes: one that occupies the entire dorsal brain, and two others occurring contralaterally just above the developing palps (Fig. 4A). A bilateral pair of cell clusters abuts the brain posterolaterally, and several neuronal processes extend between each cluster and the neighboring brain region (Fig. 4A; only one cluster is shown). These conspicuous clusters are interpreted here as the posterodorsal-most brain ganglia; the significance of their separation from the rest of the brain and the process by which it occurs is not understood. Another conspicuous feature of the hatchling head is a dorsolateral longitudinal nerve that joins the dorsal root of the CC and appears to make connections with peripheral nerves posterior to the brain (Fig. 4A).

By the late 3-chaetiger stage, the prostomium has proliferated outward from the yolk, acquiring a more three-dimensional geometry (Fig. 1C). As a corollary, the brain has expanded along the anterior-posterior axis, but it remains relatively simple, as few recognizable ganglia and cephalic nerves have formed (Fig. 4B-E). Cells intercalated by an abundance of neuronal processes characterize the brain's superior-most cortex (dorsal to the neuropile), which is referred to here as the posterordosal cortex (Fig. 4B). This part of the brain is situated superficially in the head; a distinct morphological separation between it and an overlying epidermis has apparently not yet developed. The posterodorsal-most brain ganglia reside at this level of the head, although their locations could only be approximated from the dorsal aspect examined here (Fig. 4B; note that cell proliferation in the brain and neighboring regions begins to fill the space that isolated these ganglia in hatchlings). The nuchal ganglia, the bipolar primary chemosensory neurons of the nuchal organs [64] (see below), are present in the posterior brain just inferior and slightly anterior to the posterodorsal-most brain ganglia, and are rooted in the dorsal neuropile via the nuchal nerves (Fig. 4C). The development of the nuchal system in early juveniles of *N. arenaceodentata *is congruent with the polychaete *Capitella *(distantly related to nereidids), whose nuchal system becomes recognizable by mid-larval stages [65].

Also at the dorsal level of the prostomium, a large mass of brain cells, here referred to as the anterordorsal cortex, occupies a broad medial domain between the neuropile and anterior head (Fig. 4C). Ventral to this cortex, cells near the bases of the developing antennae likely correspond to the antennal ganglia (seen definitively in later stages; see below), which are rooted in the neuropile via antennal nerves (Fig. 4D). The dense mass of cell bodies occurring at this level of the brain, between the developing antennal nerves and in front of the neuropile, is referred to here as the anteromedian cortex. Ventral to the latter, near the floor of the brain and at a level exposing the roots of the CCs, is the anteroventral cortex (Fig. 4E). Until a more sophisticated understanding of the juvenile nereidid brain is achieved, these broad divisions of the cortex may be helpful in describing, for example, domains of developmental gene expression. Small portions of the neuropile angle slightly forward at this level of the brain (Fig. 4E); most of their fibrous content probably consists of the axial palp nerves, but because other lateral cephalic nerves converge on these structures, they are referred to here as "lateral common roots".

#### 5-chaetiger stage

In 5-chaetiger juveniles, the brain's posterodorsal cortex is bilobed. The rounded edges of these lobes reach the posterior border of the prostomium and presumably house the posterodorsal-most brain ganglia (Fig. 4F). The area between the lobes, which is dorsal to the pharynx, is only sparsely populated with cells, and may not be part of the brain. By this stage distinct cilia have developed distal to each nuchal ganglion (Fig. 4G, H), yielding a more complex nuchal organ. These are likely sensory cilia of the dendrites of nuchal organ perikarya, and may thus reside in newly differentiated olfactory chambers. This labeling may also indicate differentiation of the nuchal organ supporting cells, which become ciliated, form an epidermis overlying the olfactory chambers, and generate water currents to facilitate chemosensation (see [66] and reviews by Purschke [49,64]). The antennal nerves are clearly visible coursing along the sides of the anteromedian cortex (Fig. 4H), and like the antennal nerves of other nereidids, are rooted posterolaterally in the neuropile [56]. The many smaller nerves joining the neuropile laterally (marked by dashed arcs in Fig. 4H and 4I) may represent nerves of the palp walls and the multiple fibers that condense during juvenile growth to form the adult head's tegumentary nerves, which supply a subepidermal plexus (in *A. virens*; [42]). Lastly, a pair of nascent ganglia appears to be present on the midline of the anteromedian cortex (Fig. 4H), and the 1st pair of stomatogastric nerves has developed into conspicuous structures penetrating the anteroventral cortex (Fig. 4I).

#### 9-chaetiger stage

Cells of the brain and prostomial epithelium are still generally continuous in 9-chaetiger juveniles; no obvious epidermis has formed (as seen in histologic sections of plastic-embedded specimens; data not shown). This is consistent with *H. diversicolor*, where brain ganglion cells of at least eight-segmented juveniles fill the head from the neuropile outward to the periphery [42]. By adulthood, however, the nereidid brain is internalized within the prostomium, surrounded by a fibrous neural lamella, and separated from a thick epidermis [6]. Interestingly, in the polychaete *Capitella*, brain internalization and separation from a distinct epidermis occurs much earlier: within about a week of development at 19°C — before larval metamorphosis [65,67]. The cephalic nervous system in *N. arenaceodentata *is nevertheless remarkably complex in 9-chaetiger juveniles (Fig. 5A-D). The posterior brain has developed distinct ganglia, each of which appears to be connected by its own processes to the dorsal-most portion of neuropile (Fig. 5A; note that this Z-projection is positioned approximately 27 μm below the surface of the head, thus the posterodorsal-most brain ganglia may not be visible). Identification of these immature ganglia awaits more thorough investigations of brain morphogenesis through ontogeny. Knowledge of the organization of nereidid brain ganglia is based primarily on the adults of *H. diversicolor *(e.g., [68,69]); given the possibilities of interspecific differences and spatial shifting of ganglia during ontogeny, brain anatomy of juvenile *N. arenaceodentata *may not accurately reflect that of adult *H. diversicolor*. However, it is thought that across all nereidids, ganglia in this portion of the brain (including the nuchal ganglia) are related functionally as hormone releasing centers controlling growth and sexual maturation. They accordingly contain high concentrations of neurosecretory cells, some types of which are found nowhere else in the brain (e.g., [70-73]).

Cilia of the nuchal organs have apparently lengthened beyond the cuticle by the 9-chaetiger stage (Fig. 5A), suggesting the presence of fully functional epidermal supporting cells, possibly even fully functional nuchal organs. In some 9-chaetiger juveniles, the two pairs of eyes had not yet assumed their final positions relative to one another. Such is the case for the specimen figured here, as the anterior eyes are present on the rounded sides of the head just ventral (not anterior) to the posterior eyes (Fig. 5B). The optic nerves were not detected; they likely form during later development. At the same level as the anterior eyes (i.e., Fig. 5B), the nuchal nerves and ventral portions of the nuchal ganglia are present in the posterior brain, and the anterodorsal cortex fills the anterior brain in front of the neuropile. Below this, many fine nerve fibers issue from the front of the neuropile and pass into the anteromedian cortex (medial to the antennal nerves; Fig. 5C). Some of these fibers disappear into what may be the dorsal portion of a bilateral pair of stomatogastric ganglia that abut the mid-sagittal plane. Inferiorly (in the anteroventral cortex), these ganglia are more apparent, and minute connections between them and the 1st pair of stomatogastric nerves are present where the nerves arc posteriorly toward the pharynx (Fig. 5D). The roots of the 2nd pair of stomatogastric nerves, unlike the 1st pair, lack defined ganglia in front of the neuropile; this may be why they do not project forward before arcing posteriorly toward the pharynx (Fig. 5D, arrowheads).

#### Antennal ganglia and corpora pedunculata

The neuropile of 9-chaetiger juveniles expands considerably at its sides and displays an outward radiation of nerves at the level of the anteromedian cortex (Fig. 5C). Most of these nerves join the neuropile laterally (dashed arcs), and as described above for 5-chaetiger juveniles, probably represent nerves of the palp walls and other tegumentary nerves of the head. The largest nerves of this radiation extend anteriorly from the neuropile. First, each antennal nerve courses toward an ipsilateral ganglion located just posterior to the antenna (Fig. 5C). To the authors' knowledge, these antennal ganglia, which appear to receive the central processes of the antennal sensory organs (Fig. 2G and 5E), have not been described for nereidids. It is not clear from the data presented here what function they serve, but they may contain interneurons that relay signals from the antennal sensory organs to the brain (see Smith's [28] Fig. 23). Second, a bilateral pair of nerves joins the neuropile lateral to the antennal nerves (Fig. 5C, E). The pronounced ganglia at their distal ends are interpreted here as the rudimentary dorsal masses of the corpora pedunculata, as they exhibit several features of adult nereidid corpora pedunculata [3,74]: 1) they reside semi-dorsally in the brain, developing in a position between the antennal and axial palp nerves, 2) they show intense nuclear staining, suggesting they contain globuli cells (minute, tightly packed, and chromatin-rich neurons), and 3) in the latest juvenile stage examined (20 chaetigers), the nerves rooting them in the neuropile develop into thick stalks in close proximity to the palp nerve roots and medial to the anterior eyes (data not shown).

At the level of the CCs and their roots (Fig. 5D), evidence is found for a second, ventral pair of masses belonging to the corpora pedunculata. This result is consistent with Hamaker [27], who found that the corpora pedunculata (which he called "mushroom bodies" because of their similarity to structures of the same name in arthropod brains) of *A. virens *consist of both dorsal and ventral masses. In 9-chaetiger juveniles of *N. arenaceodentata*, these nascent ventral masses are barely compact enough to be recognized, but their presence is evident by the distinct nerves connecting them to the anterior neuropile (Fig. 5D). These nerves are at slight angles to, and occur just below, the nerves of the dorsal masses of the corpora pedunculata. The nerves of both masses are joined proximally (just medial to the palp nerves), indicating they share a common root location (Fig. 5E; here, the dorsal masses overlie the ventral masses, obscuring their view). In 20-chaetiger juveniles, the corpora pedunculata of *N. arenaceodentata *occupy a larger proportion of the prostomium, and are positioned more anteriorly than the adult corpora pedunculata of other nereidid species (e.g., *H. diversicolor*; [74]) (Fig. 5F).

#### Langdon's organs and commissural ganglia

Langdon's organs are evident just lateral to the ventral masses of the corpora pedunculata (Fig. 5D, E). Langdon [50], studying *A. virens*, described the microanatomy of these elongate sensory organs, which reside in the dorsal prostomium between the antennae and palps; Gilpin-Brown [42] provided their name. Although the stimulus to which these organs respond is unknown, they are a common feature among nereidids, having been found in *H. diversicolor *[68,75], *Perinereis cultrifera *[76], and *Nereis pelagica *[56]. The sensory-cell processes within the Langdon's organs of *N. arenaceodentata *juveniles were not readily detectable, but patches of intense acetylated α-tubulin immunoreactivity are present at their anterior termini (Fig. 5D, E). These likely correspond to the subcuticular, bunched terminal endings of the sensory cells' peripheral processes. In 20-chaetiger juveniles, these bunched peripheral processes are located immediately in front of the ventral masses of the corpora pedunculata, at the anterolateral edge of the prostomium, precisely as Langdon [50] drew them (Fig. 5F; her Fig. 28).

Lastly, by the 9-chaetiger stage, faint nerve fibers emanate laterally from the dorsal roots of the CCs and from the confluences of the dorsal and ventral roots of the CCs (Fig. 5D). These may be the constituent fibers of developing ganglia that have been identified at the aforesaid locations in the adults of several nereidid genera. First, Holmgren's cerebral commissural ganglia abut the dorsolateral sides of the dorsal roots, and lie laterally in the prostomium just below the anterior eyes [27,68,76]. Despite their proximity to the eyes, these ganglia probably do not function in vision, as their fibers are directed only toward ganglia found at the second location: Hamaker's commissural ganglia [27,56]. These reside at the junctions of the roots of the CCs. Issuing from Hamaker's commissural ganglia are three types of nerves: a stomatogastric nerve, a tegumentary nerve, and a small "extra root" of the CC [56]. These nerves are not evident in Fig. 5D because neither the stomatogastric nor the tegumentary nerve appears to have developed, and the extra root is obscured by the overlying dorsal root. The stomatogastric nerve, however, is clearly developed by the 12-chaetiger stage (Fig. 2G, third arrow down).

### Directions for prospective research

Continued research on the morphology and development of polychaete nervous systems will be valuable for achieving a deeper understanding of bilaterian evolution. Several relevant issues are outlined here. First, further fine-scale morphologic analyses are needed to determine the topographic origins and spatial dynamics of developing brain ganglia and cephalic sense organs. Investigations of wholemount heads using CLSM and antibodies to a variety of neural markers will be informative, particularly when the labeling is analyzed by modern 3D reconstruction software (e.g., [77]). Use of such software to reconstruct internal head anatomy from histologic serial sections (for light or transmission electron microscopy) will be equally useful (e.g., [78]), as will studies incorporating immunohistochemistry and in situ hybridization for neural-patterning genes. Extensive knowledge of cephalic neuroanatomy through ontogeny will enable more accurate identification of gene expression domains, lending support to hypotheses of gene function and to arguments for, or against, various homologies in different taxa. For example, if the development of polychaete corpora pedunculata is accompanied by expression of the same genes deployed during the development of arthropod mushroom bodies, then homology of these brain structures, which has been proposed on morphological grounds [79], would be supported. In addition, polychaete nuchal organs and vertebrate olfactory mucosa share several ultrastructural similarities [66]; comparative developmental genetics of these sensory structures may indicate an evolutionary relationship such as cell-type homology.

A second intriguing avenue for future research lies in the descriptive morphology and development of the inadequately explored stomatogastric nervous systems (SNSs) of polychaetes. Comparisons both within Annelida and to other bilaterian phyla may reveal patterns of SNS development and functionality, as well as characters potentially useful for phylogeny reconstruction. In terms of phylogeny, relevant characters may derive from the number and position of stomatogastric nerve roots, longitudinal nerves, nerve rings, ganglia, and motor axon termini. Relating to development and functionality, it will be interesting to examine whether developmental-genetic mechanisms are shared between the SNSs of annelids and other bilaterian phyla, and whether annelid SNSs, like those of arthropods, are capable of autonomously controlling multiple types of rhythmic foregut movement. Among arthropods, much is known about SNS development in insects [80], and the crustacean SNS is a leading model in the study of peripheral neuronal circuits and their control over rhythmic behaviors [81]. In the lobster foregut, for example, separate stomatogastric circuits control gastric chewing, pyloric peristalsis, and water swallowing (to increase internal body pressure for ecdysis) [82]. The foreguts of errant polychaetes also exhibit multiple behaviors. In nereidids, for example, the isolated esophagus spontaneously contracts with a complex, rhythmic pattern [83], and different patterns of pharyngeal protrusion and jaw movement appear to correlate with feeding, burrowing, and fighting (CJW, unpublished observations). Thus, it is reasonable to anticipate that morphological and physiological analyses will reveal the existence of SNS circuits that regulate the varied motor responses of polychaete foreguts.

Third, the organization of motor innervation of polychaete somatic muscles is poorly known and controversial. In nereidids and polynoids, very few (approximately nine) motor neurons have been found in each VNC ganglion [28,84]. These surprisingly small cell counts have led to several hypotheses concerning motor architecture [28]: 1) efferent signals from a single VNC motor neuron may be routed directly to a set of functionally related muscles via one highly branched axon; 2) axons of the VNC motor neurons may synapse with a system of peripherally located, second-order motor neurons, such as within the parapodial ganglion, that divide the primary efferent signal and relay it to multiple muscles; and 3) reflex circuits may be present in the PNS that effect responses to sensory stimuli without communication with the CNS. In support of hypothesis 1, two VNC motor neurons of the polynoid *Harmothoe *appear to branch multiple times upon leaving the cord, with each major branch coursing directly to a separate muscle [84]. Providing support for the presence of motor neurons in the parapodial ganglia (hypothesis 2, partially), and for peripheral reflex circuits independent of the CNS (hypothesis 3), isolated parapodia of *N. brandti *responded with repeatable patterns of contraction upon chemical and tactile stimulation, with no response observed in parapodial isolates lacking a ganglion [55]. Dorsett [51] observed another neuromuscular arrangement for what he called the parapodial retractor muscle in nereidids. Basically, three motor axons emerged from the VNC (they were not part of any segmental nerve) and terminated directly on this muscle. He therefore de-emphasized the role of peripheral motor neurons in polychaetes, and concluded that one fast, one slow, and one inhibitor motor neuron triply innervate this muscle, similar to certain arthropod muscles. Mettam [45], however, downplayed the importance of polyneuronal innervation. He pointed out that Dorsett's [51] parapodial retractor is actually three muscles: the posterior parapodial obliques. These function in rapid parapodial deflection during swimming and thus do not require slow motor innervation [45]. Efforts to establish a clearer understanding of polychaete parapodial ganglia and motor-neuronal arrangements for somatic musculature will benefit from the fine-scale resolution and efficiency of CLSM. Retrograde labeling with DiI [85,86] and immunohistochemistry for various myoactive substances such as serotonin [87], Polychaete Excitatory Peptide [88], and acetylcholine [5] (visualized with antibodies to choline acetyltranferase [89]) should prove effective in locating motor-neuronal perikarya and mapping their axonal pathways to muscle.

## Conclusions

The direct-developing juveniles of *N. arenaceodentata *appear to have lost all essential larval features. Their developing neuroanatomy accords well with the organization of adult nereidid nervous systems. Many elements of the cephalic nervous system (e.g., stomatogastric nerves and ganglia, nuchal organs, sensory appendages, and corpora pedunculata) become morphologically distinguishable during early juvenile stages, approximately two weeks before emergence from the parental tube and the onset of feeding. The difference in the timing of brain internalization noted for *Capitella *versus nereidids indicates divergent mechanisms of prostomial development. *Capitella*'s early internalization and the apparent posterior displacement of its brain (see Meyer and Seaver's [67] Fig. 1K) may have evolved as a means of protecting the brain from the physical impacts of this worm's actively burrowing lifestyle. Similar arguments have been made for clitellate annelids (e.g., [29]).

In terms of peripheral trunk innervation, Smith's [28] influential study apparently does not account for variation among the nereidids *Platynereis dumerilii*, *Hediste diversicolor*, and *Alitta virens*. The single arrangement of parapodial nerves he presented for these genera contrasts with Hamaker's [27] description for *A. virens*, which is very similar to the pattern found here for *N. arenaceodentata*. Furthermore, no evidence was found here for Smith's [28] lateral nerves. Given Hamaker's [27] firm conviction that no such nerves exist in *A. virens*, it seems that lateral nerves are another variable character among nereidids. Taxonomic bias is another reason to expect greater variation in nereidid neuroanatomy than previously appreciated, as neural morphology is yet to be investigated in most of the major nereidid subgroups (see the phylogeny of Santos et al. [43]).

Immunohistochemistry combined with CLSM is an effective approach for analyzing peripheral nervous systems [53]. Accordingly, the current study reveals previously undescribed aspects of parapodial muscle innervation and peripheral nerve interconnections. First, dorsomedial branches of the parapodial nerves pn1 and pn3 respectively innervate the anterior and posterior sets of dorsal parapodial muscles; pn3 appears to also innervate the parapodial levator muscle. Second, acicular muscles, including the neuropodial protractor, are innervated at their origins around the acicular base by acicula-associated nerves, which traverse most of the parapodium's width in a distal-to-proximal direction to reach their termini. Third, a segmental peripheral plexus located ventrolaterally appears to integrate fibers from the 4th segmental nerve, the parapodial nerve pn4, and a nerve that extends from the posterodorsal parapodium. Fourth, a peripheral interconnection occurs between the parpodial nerve pn3 and a nerve that courses dorsomedially but whose terminus in that direction is uncertain.

Finally, direct-developing nereidids like *N. arenaceodentata *serve as interesting comparisons to indirect-developing nereidids like *P. dumerilii *and *A. virens*. Early developmental comparisons involving, for example, morphology or gene expression may shed light on the mechanisms underlying evolutionary loss of larvae. Additionally, several features associated with *N. arenaceodentata*'s direct development make it an experimentally tractable animal. For example, its large eggs, around 450 μm in diameter, are roughly 15 times larger than *P. dumerilii *and *A. virens *eggs, and may thus be easier to manipulate. With no feeding requirement, large broods of around 400 synchronously developing individuals can be easily harvested from the parental tube up through the 20-chaetiger stage.

## Methods

### Animal culturing

An initial laboratory population of *Neanthes arenaceodentata *was established at UCLA using individuals from Dr. Donald Reish's long-standing "Los Angeles Harbor" colony at California State University-Long Beach. Due to poor reproductive success, the initial population crashed and was replaced with worms isolated from a local natural population (Ballona Lagoon; Venice, CA). Specimens used for this study came from both the old and new UCLA populations. Adult worms were kept in an air-conditioned (≈21°C), moderately sunlit room in a 5-gallon aquarium with 5-μM filtered seawater (salinity ≈32 ppt) and a layer of fine sand that measured ≈1 cm deep. A Whisper Power Filter 10 circulated the water. Adults were fed 3 times weekly a 2:1 mixture of finely chopped *Ulva clathrata *(collected from the Ballona Lagoon, rinsed in filtered seawater, and stored frozen) and crushed saltwater fish flakes; enough food was used to completely cover the sand. Feeding proceeded for 4-6 hours, during which the filter was turned off. Afterward, the seawater and remaining food were siphoned away and replaced with clean seawater. Reproductive pairs were chosen [39,90] and placed in 1-gallon jars fitted with bubblers. Seawater in the jars was exchanged once weekly, at which time the pair was fed only chopped *Ulva*. Extra *Ulva *was provided for tube construction. Juveniles were harvested from parental tubes and either fixed for analysis (see below) or transferred to a 5-gallon aquarium for population perpetuation. Near the onset of feeding, the latter worms were given a 1:1 mixture of finely ground Gerber Mixed Grain Cereal For Baby and saltwater fish flakes. The adult diet was provided when juvenile tubes could be seen clearly with the naked eye. At this point, sand was added incrementally as the juveniles aged.

### Specimen fixation and storage

Worms were relaxed in 7.3% MgCl _2 _until no movement was observed, and then fixed with 4% formaldehyde in seawater overnight at 4°C. Fixative was washed away with three 5-min washes in phosphate buffered saline plus 0.1% Tween 20 (PBTw), followed by two 5-min washes in water plus 0.1% Tween 20. Specimens were then dehydrated with 5-min rinses in a series of increasing concentrations of methanol (25%, 50%, 75%, 2× 100%), and finally stored at -20°C in 100% methanol. Prior to use, specimens were rehydrated with 5-min rinses in decreasing concentrations of methanol in PBTw (75%, 50%, 25%, 3× 100% PBTw). For F-actin labeling, specimens were fixed as above, but with 4% paraformaldehyde. They were then washed 5× in PBTw, and stored at 4°C in PBTw with 1 mg/mL sodium azide.

### Scanning electron microscopy

Specimens were taken from storage and rehydrated as above, but the methanol solutions were in 0.1M sodium cacodylate buffer plus 0.1% Tween 20 (SCTw). Additional fixation was done for 2 hours with parafomraldehyde/glutaraldehyde, 2.5% each, in SCTw. Following three 5-min washes in SCTw, specimens were postfixed with 1% osmium tetroxide in SCTw for 1 hour. After 3 more 5-min washes in SCTw, the specimens were dehydrated with 5-min rinses in increasing concentrations of ethanol (25%, 50%, 70%, 80%, 90%, 3× 100%). Specimens were then rinsed for 10 min in 1:1 ethanol:HMDS (hexamethyldisilizane), rinsed 2× 10 min in 100% HMDS, air dried for 2 hours, affixed to double-coated carbon tabs on aluminum stubs, sputter-coated with gold, and visualized with a JEOL JSM-6700F field emission scanning electron microscope. Specimens processed for SEM experienced approximately 30% shrinkage relative to those fixed only in formaldehyde.

### Fluorescent labeling, microscopy, and image analysis

After rehydration, whole specimens were digested with 20 μg/mL Proteinase K in PBTw (dissected specimens were not subjected to this treatment). This lasted 5 min for juveniles with up to 4 chaetigers, 8 min for 5- to 10-chaetiger juveniles, and at least 12 min for all older juveniles. Note that, despite the longer proteinase incubation times for older specimens (12- and 20-chaetigers), sufficient labeling intensity was difficult to achieve and varied considerably among specimens. The proteinase activity was terminated with 3 rinses (2 quick and 1 for 5-min) in freshly prepared 2 mg/mL glycine in PBTw, followed by 3 more rinses in PBTw. The specimens were then refixed with 4% formaldehyde in PBTw for 30 min, followed by five 5-min washes in PBTw. Further permeablization was accomplished with a 1-hour wash in phosphate buffered saline plus 0.1% Triton X-100 (PBTr). Non-specific binding sites were blocked for 1 hour with 2.5 mg/mL bovine serum albumin (BSA) in PBTr. The primary antibodies, monoclonal anti-acetylated α-tubulin (Sigma-Aldrich), were diluted 1:800 in PBTr+BSA, and applied for 18 hours at 4°C. This was followed by 1 hour of washing with at least 4 changes of PBTr, and 1 hour of blocking in PBTr+BSA. The fluorescent secondary antibodies, Alexa Fluor 568 (or 488) goat anti-mouse IgG (Invitrogen), were diluted 1:200 in PBTr+BSA and applied for 18 hours at 4°C. Following 1 hour of washing in PBTr and then 5 quick washes in phosphate buffered saline (PBS), the specimens were cleared and mounted in Vectashield (Vector Laboratories) containing 1:500 TOTO-3 iodide (Invitrogen), a fluorescent nuclear stain.

Following immunolabeling, some specimens were labeled for F-actin. To achieve this, an aliquot of Alexa Fluor 568 phalloidin (Invitrogen) was dried by spinning in a lidless centrifuge and resuspended to 1:40 in PBTr+BSA. Specimens were incubated in this solution for 1 hour, washed 3× 5 min in PBTr, washed 5× quickly in PBS, and mounted in Vectashield plus 1:500 TOTO-3 iodide.

The Leica TCS-SP1 MP system for confocal laser scanning microscopy was used to visualize fluorescent labels. Image adjustment and production of Z-projections of optical sections was accomplished with ImageJ software.

## Competing interests

The authors declare that they have no competing interests.

## Authors' contributions

CJW collected and cultured *Neanthes*, carried out all aspects of specimen preparation, performed the CLSM, and drafted the manuscript. CJW conceived the study with DKJ, and DKJ contributed to writing of the manuscript. JEV scanned the specimens for SEM. All authors read and approved the final manuscript.
